# Transcutaneous Auricular Vagus Nerve Stimulation Protects Endotoxemic Rat from Lipopolysaccharide-Induced Inflammation

**DOI:** 10.1155/2012/627023

**Published:** 2012-12-29

**Authors:** Yu Xue Zhao, Wei He, Xiang Hong Jing, Jun Ling Liu, Pei Jing Rong, Hui Ben, Kun Liu, Bing Zhu

**Affiliations:** Institute of Acupuncture and Moxibustion, China Academy of Chinese Medical Sciences, 16 Nanxiaojie Street, Dongzhimen Nei, Beijing 100700, China

## Abstract

*Background*. Transcutaneous auricular vagus nerve stimulation (ta-VNS) could evoke parasympathetic activities via activating the brainstem autonomic nuclei, similar to the effects that are produced after vagus nerve stimulation (VNS). VNS modulates immune function through activating the cholinergic anti-inflammatory pathway. *Methods*. VNS, ta-VNS, or transcutaneous electrical acupoint stimulation (TEAS) on ST36 was performed to modulate the inflammatory response. The concentration of serum proinflammatory cytokines and tissue NF-kappa B p65 (NF-**κ**B p65) were detected in endotoxaemia affected anesthetized rats. *Results*. Similar to the effect of VNS, ta-VNS suppressed the serum proinflammatory cytokines levels, such as tumour necrosis factor-alpha (TNF-**α**), interleukin-1 beta (IL-1**β**), and interleukin-6 (IL-6) as well as NF-kappa B p65 expressions of lung tissues. ST36 stimulation also decreases LPS-induced high TNF-**α** level and NF-**κ**B signal, but it did not restrain proinflammatory cytokine IL-1**β** and IL-6. Neither ta-VNS nor ST36 stimulation could suppress LPS-induced TNF-**α** and NF-**κ**B after vagotomy or with **α**7nAChR antagonist injection. *Conclusions*. The present paper demonstrated that ta-VNS could be utilized to suppress LPS-induced inflammatory responses via **α**7nAChR-mediated cholinergic anti-inflammatory pathway.

## 1. Introduction 

Inflammation is a local, protective response to microbial invasion or injury, which must be fine-tuned and regulated precisely [1]. Several potent cytokines including tumour necrosis factor-alpha (TNF-*α*), interleukin-1 beta (IL-1*β*), interleukin-6 (IL-6), interleukin-10 (IL-10), and transforming growth factor-beta (TGF-*β*) are produced by activated macrophages and other immune cells, as necessary and sufficient mediators involved in local and systemic inflammation [2, 3]. Overproduction of cytokines leads to systematic inflammation and tissue injury. If the overproduced cytokines spread into the bloodstream, dangerous inflammatory responses will be induced. In the past ten years, abundant studies have been focused on “the cholinergic anti-inflammatory pathway,” namely, the efferent vagus nerve which inhibits proinflammatory cytokine production and protects against systemic inflammation via a *α*7nAChR-dependent pathway [4]. Vagus nerve stimulation (VNS) prevents the occurrence and development of inflammation effectively via activating the cholinergic anti-inflammatory pathway. VNS and acetylcholine (Ach) attenuated the release of cytokines significantly and improved survival in lethal endotoxemia or sepsis models [5, 6]. For instance, in a rat model of lethal endotoxamia, electrical stimulation of the efferent vagus nerve decreases serum and hepatic TNF levels [6]. Moreover, VNS inhibited all lipopolysaccharide- (LPS-) induced procoagulant responses strongly, attenuated the fibrinolytic response more modestly, and improved hepatic Ach levels significantly in endotoxemia rats [7]. VNS attenuated the LPS-induced increases of the plasma and splenic proinflammatory cytokines, rather than influencing the anti-inflammatory cytokine IL-10 [8]. In addition, activation of this neural immune-modulatory pathway by electrical stimulation of vagus nerve also protects animals from various circumstances, such as ischemia-reperfusion injury, hypovolemic hemorrhagic shock, heart failure, and myocardial ischemia/reperfusion [9–12].

It was demonstrated that the transcutaneous auricular vagus nerve stimulation (ta-VNS) induced a series of parasympathetic activities [13–17]. Auricular branch of vagus nerve is a special vagal branch that innervates the body surface which could not be found on the other parts of the body [18], mainly innervating the cymba conchae and cavum conchae within the auricle. Our previous studies indicated that there is an intimate connection between auricular concha, the nucleus tractus solitarius (NTS), dorsal motor nucleus of the vagus nerve (DMN), and vagus nerve, which constructs the pathway of the auricular-vagal reflex. Accordingly, there might be some kinds of connection between auricular concha and efferent vagus nerve. In the present study, we reasoned that ta-VNS may have a role in activating the vagus nerve-based cholinergic anti-inflammatory pathway. Here, the effect of ta-VNS on proinflammatory cytokines and NF-kappa B p65 was explored to clarify the mechanism of ta-VNS underlying regulating inflammatory diseases.

## 2. Materials and Methods

### 2.1. Animals

Male Sprague Dawley rats (12 weeks old) were used in the present study, weighing 275–350 g, supplied by China Academy of Military Science. Rats were housed in groups of 5-6 in standard polycarbonate cages at ambient temperature (22°C) and allowed access to food and water* ad libitum. *Lights were set to an automated 07:00 on and 19:00 off light-dark cycle, and all animal experiments were done between 08:00 and 11:00 a.m. Rats received care consistent with *the National Institutes of Health Guide for the Care and use of Laboratory Animals*, and the experiments were conducted in accordance with a protocol approved by the Institutional Animal Care and Use Committee of China Academy of Chinese Medical Sciences. 

### 2.2. Experimental Protocols

The present study consisted of 4 main parts. (1) The first detects the effect of ta-VNS, VNS, or transcutaneous electrical acupoint stimulation (TEAS) on ST36 on LPS-induced serum cytokine response. In this part, rats were randomly divided into 5 groups of twelve each: (a) saline-treated animals (NS), (b) endotoxemia model rats (LPS), (c) endotoxemia model rats receiving treatment of ta-VNS (ta-VNS), (d) endotoxemia model rats receiving treatment of VNS (VNS) and (e) endotoxemia model rats received treatment of TEAS on ST36 (ST36). (2) The second detects the effect of ta-VNS, VNS, or TEAS on ST36 on LPS-induced pulmonary NF-*κ*B p65 expression. In this part, rats were randomly divided into 6 groups, which consisted of the 5 groups aforementioned, and a group of saline-treated animals received treatment of ta-VNS (NS+ta-VNS). (3) The third observes the effect after vagotomy (VGX). In this part, rats were randomly divided into 4 groups: (a) saline-treated animals (NS), (b) endotoxemia model rats (LPS), (c) ta-VNS-treated animals following vagotomy (VGX+LPS+ta-VNS), and (d) TEAS-treated animals following vagotomy (VGX+LPS+ST36). (4) The fourth observes the effect after *α*7nAChR antagonist injection. Rats were randomly divided into 4 groups: (a) saline-treated animals (NS), (b) endotoxemia model rats (LPS), (c) ta-VNS-treated animals following *α*-bungarotoxin (*α*-BGT+LPS+ta-VNS), and (d) TEAS-treated animals following *α*-bungarotoxin (*α*-BGT+LPS+ST36).

### 2.3. Endotoxemia Model

**Figure 1:**
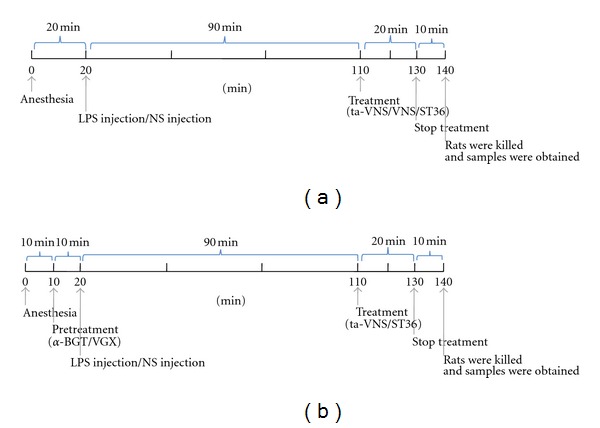
The time flow chart indicates the precise time for various operations in the present study, from the time point of anesthetic injection to the time point for sampling. (a) Twenty minutes after anesthesia, rats were injected intravenously with LPS or NS. One and a half hour after modeling, treatment (ta-VNS, VNS, or TEAS on ST36) was performed for twenty minutes. Two hours after LPS injection, rats were killed, and samples were collected. (b) Ten minutes after anesthesia, administration of *α*-BGT or vagotomy was performed. The rest of the operations were the same with the time flow in (a).

LPS is an endotoxin derived from cell wall of gram-negative bacteria, and systemic injection of LPS results in various symptoms of bacterial infection including fever and inflammation [19]. Rats were injected intravenously with lipopolysaccharide (LPS, *Escherichia coli* 0111:B4; Sigma, 5 mg/kg), dissolved in sterile, pyrogen-free saline that was sonicated for 30 minutes immediately before use. Rats (*n* = 12 per group) were killed 2 hours after LPS injection (Figure 1), and the blood was collected from abdominal aorta, allowed to clot for 2 hours at room temperature, and then centrifuged at room temperature for 15 minutes at 2000 rpm. Serum samples were stored at −20°C before cytokine analysis. Lung samples were rapidly excised, rinsed of blood with normal saline, placed into liquid nitrogen immediately and then frozen and stored at −80°C till measurement of NF-*κ*B p65 expression. 

### 2.4. Electrical Vagus Nerve Stimulation

Rats were anaesthetized with urethane (1 g/kg, intraperitoneally). A midline cervical incision was made to expose the left cervical branch of the vagus nerve. The left carotid sheath was isolated. After blunt preparation, the left vagus nerve trunk was carefully freed from surrounding tissue, separated from the carotid artery trunks, and placed on a custom-made bipolar platinum electrode connected via an isolation unit to a stimulator (SEN-7203, Nihon Kohden). All the exposed nerves were protected from dehydration by covering warm paraffin mineral oil tampons [6]. One and a half hour after LPS administration, constant electrical current stimuli with parameter of 1 mA, 10 Hz, 1 ms were turned on for 20 min (Figure 1(a)). 

### 2.5. Transcutaneous Electrical Acupoint Stimulation (TEAS) on ST36

Transcutaneous surface electrodes were placed bilaterally on the depilatory rat skin at Zusanli (ST36). The ST36 points are located at 5 mm lateral to the anterior tubercle of the tibia and 10 mm below the knee joints. Bilateral surface electrodes at hind limbs were connected via an isolation unit to a stimulator (SEN-7203, Nihon Kohden), and the points were stimulated with the same parameter aforementioned. One and a half hour after LPS administration, the stimulation started and lasted for 20 min (Figure 1). Stimulation intensity was adjusted to a level that elicited a slight muscle twitch at the stimulated site and was limited to a maximum of 1 mA to minimize animal discomfort.

### 2.6. Transcutaneous Auricular Vagus Nerve Stimulation (ta-VNS)

Transcutaneous surface electrodes were placed bilaterally on the auricular concha, which mainly includes cymba conchae and cavum conchae of the auricular. Bilateral surface electrodes at auricular concha were connected via an isolation unit to a stimulator (SEN-7203, Nihon Kohden), and the auricular conchae on both sides were stimulated with the same parameter. One and a half hour after LPS administration, the stimulation started and lasted for 20 min (Figure 1). Stimulation intensity was adjusted to a level that elicited a slight twitch of the auricle and was limited to maximum of 1 mA to minimize animal discomfort.

### 2.7. Vagotomy

Vagotomy was performed before LPS administration (Figure 1(b)). In vagotomized animals, following a ventral cervical midline incision, bilateral vagus trunks were exposed and separated from the common carotid artery, ligated with a 4-0 silk suture. 

### 2.8. Administration of *α*7nAChR Antagonist

The specific *α*7nAChR antagonist *α*-bungarotoxin (*α*-BGT) was obtained from Alexis Biochemicals Corporation (San Diego, CA, USA). The drug was administered intravenously at a dose of 1 *μ*g/kg before LPS administration [20] (Figure 1(b)).

### 2.9. Cytokine Analysis

Abdominal aortic blood was collected two hours after LPS administration, allowed to clot for 2 h at room temperature, and centrifuged for 20 min at 2500 rpm. Serum TNF-*α*, IL-1*β*, and IL-6 concentrations were analyzed, respectively, by TNF-*α*, IL-1*β*, and IL-6 ELISA kits (R&D Systems) following the manufacturer's instructions.

### 2.10. Western Blot

Procedures of western blot analysis were followed as described previously [21]. Protein samples denatured in SDS sample buffer (125 mmol/L Tris-HCl, pH 6.8, 50% glycerol, 2% SDS, 5% mercaptoethanol, and 0.01% bromophenol blue) were subjected to SDS-PAGE and blotted onto Immobilon-FL transfer membrane (Millipore). Blotted membranes were blocked with 5% skim milk in Tris-buffered saline containing 0.05% Tween-20 for 2 hours and were subsequently incubated with rabbit anti-human-NF-*κ*B (p65) (diluted 1/200; Santa Cruz Biotechnology Inc., CA, USA) overnight at 4°C. After three washes in Tris-buffered saline containing 0.05% Tween 20, the membranes were incubated with an anti-rabbit IgG antibody-HRP (diluted 1/4000; Santa Cruz Biotechnology Inc., CA, USA) for 1 hour. Quantification of western blots was performed by the Odyssey infrared imaging system (Li-Cor Biosciences) to detect protein expression. 

### 2.11. NF-*κ*B Immune-Histochemistry

NF-*κ*B p65 immunohistochemistry staining was performed as described previously [22] to evaluate the lung tissues inflammatory response. Briefly, tissue sections were deparaffinized with xylene and rehydrated through graded series of alcohols. Tissue sections were rinsed in PBS, pretreated with citrate buffer at 93°C, blocked with PBS containing 2% BSA, and then incubated with a primary antibody reactive against rabbit-activated p65 subunit of NF-*κ*B (Santa Cruz Biotechnology Inc, Santa Cruz, CA, USA). Washed sections were incubated for 10 min with secondary goat anti-rabbit IgG biotin. The reaction product was visualized with DAB chromogenic agent. The sections were counterstained with hematoxylin stain. Slides were analysed on a light microscope (Olympus BX60) using an ImagePro Plus Imaging System (Universal Imaging).

### 2.12. Statistical Analysis

All the data in the present study were expressed as means ± SEM and analyzed by one-way ANOVA with SPSS software. The two-tailed Student's *t*-test was used to compare mean values between two groups. *P*values < 0.05 were considered significant.

## 3. Results

### 3.1. Cytokine Levels in the Serum

**Figure 2:**
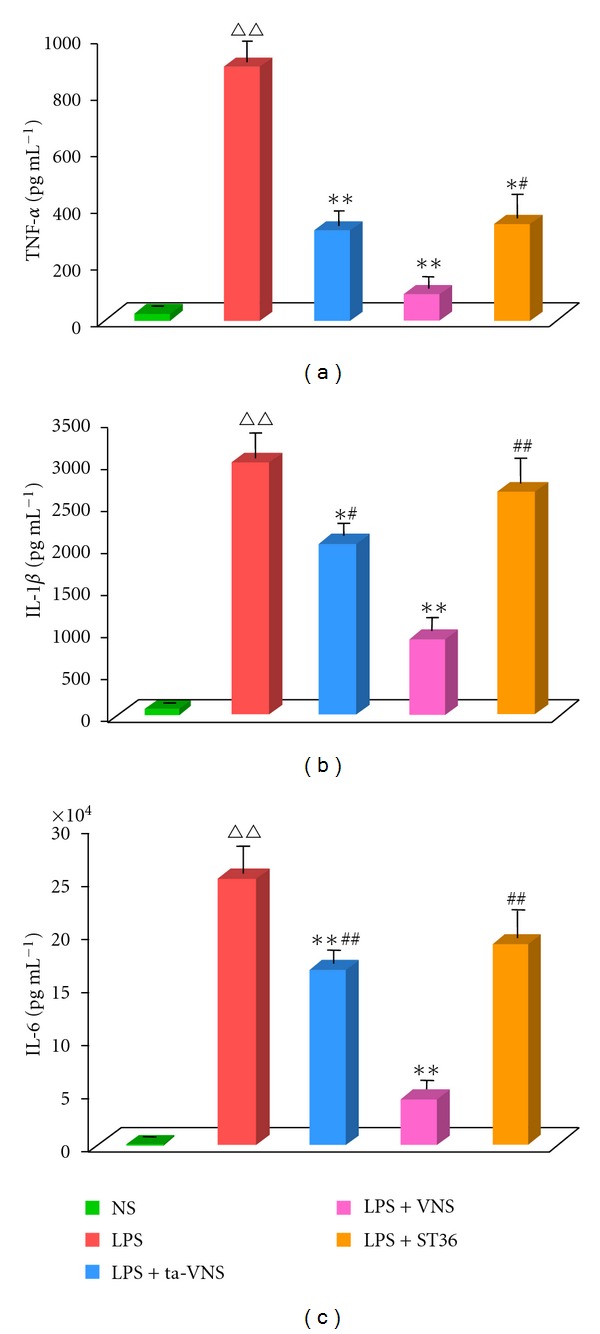
Vagus nerve stimulation (VNS) or transcutaneous auricular vagus nerve stimulation (ta-VNS) attenuates the LPS-induced serum cytokine (TNF-*α*, IL-1*β*, and IL-6) response. TEAS on ST36 inhibited TNF-*α* level significantly. Serum TNF-*α* (a), IL-1*β* (b), and IL-6 (c) contents were measured by ELISA. The columns represent mean ± SEM for 12 animals in each group. ^∆∆^ *P* < 0.01 versus the normal saline (NS) group; *P < 0.05 versus LPS group (LPS); **P < 0.01 versus LPS group (LPS); ^#^ *P* < 0.05 versus LPS+VNS group; ^##^ *P* < 0.01 versus LPS+VNS group.

LPS evoked an inflammatory response characterized by the upregulation of cytokine expressions. After systemic administration of LPS (5 mg/kg, i.v.), TNF-*α* (Figure 2(a)), IL-1*β* (Figure 2(b)), and IL-6 (Figure 2(c)) increased significantly in sera. Both electrical VNS and ta-VNS strongly inhibited LPS-induced proinflammatory cytokine concentrations including TNF-*α*, IL-1*β*, and IL-6 (*n* = 12, *P* < 0.01, *P* < 0.05, resp.). TEAS of ST36 lowered serum TNF-*α* level (*n* = 12, *P* < 0.05) in endotoxemic rats but failed to significantly alter serum IL-1*β* and serum IL-6 levels. 

### 3.2. The Effect of ta-VNS or TEAS on ST36 on Serum TNF-*α* Level Was Blocked by *α*-BGT Administration

**Figure 3:**
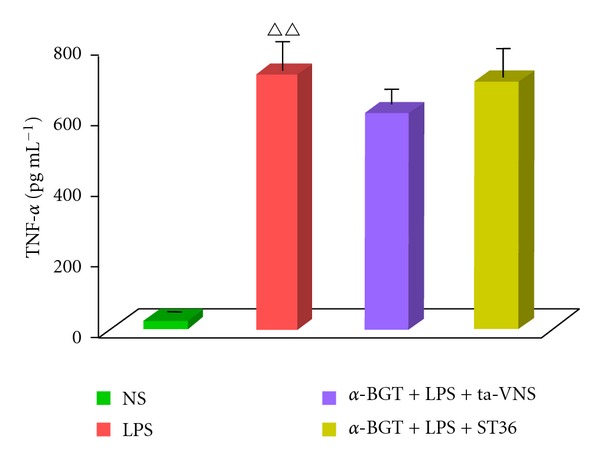
ta-VNS or TEAS on ST36 with *α*-bungarotoxin (*α*-BGT) administration fails to inhibit the LPS-induced serum TNF-*α* response. Serum TNF-*α* concentrations were measured by ELISA. Data are expressed as mean ± SEM (*n* = 12 per group). ^∆∆^ *P* < 0.01 versus the normal saline (NS) group.

The above results showed that ta-VNS has similar effects to VNS on cytokine levels. Previous studies show that VNS-activated “cholinergic anti-inflammatory pathway” regulates systemic inflammatory responses via *α*7nAChR, hereby ta-VNS may have the same effect. To test this hypothesis, we pretreated animals with the *α*7nAChR antagonist *α*-BGT. LPS injection induced profound rise in the concentration of serum TNF-*α*. Either ta-VNS or TEAS on ST36 failed to inhibit TNF-*α* level after *α*-BGT administration (Figure 3).

### 3.3. Effect of ta-VNS or TEAS on ST36 on Serum TNF-*α* Was Blunted by Vagotomy

**Figure 4:**
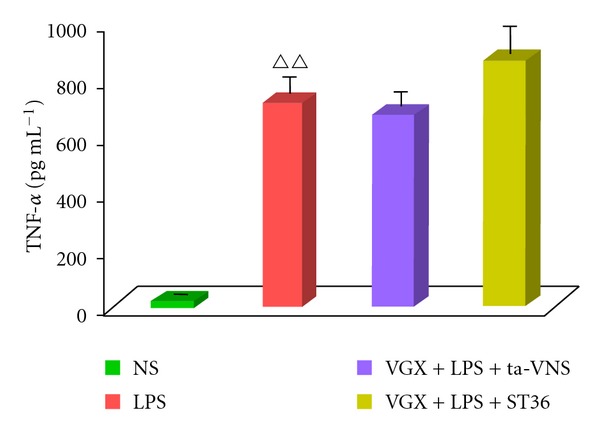
ta-VNS or ST36 stimulation with bilateral cervical vagotomy (VGX) fails to inhibit the LPS-induced serum TNF-*α* response. TNF-*α* amounts were measured by ELISA. Data are expressed as mean ± SEM (*n* = 12 per group). ^∆∆^ *P* < 0.01 versus the normal saline (NS) group.

To examine the mechanism of ta-VNS in “cholinergic anti-inflammatory pathway,” we pretreated animals with vagotomy. The result indicated that intravenous injection of LPS elicited a rapid raise of TNF-*α* level. Neither ta-VNS nor TEAS on ST36 was effective on inhibiting TNF-*α* level after vagotomy (Figure 4). 

### 3.4. NF-Kappa B p65 Expressions in Lung Tissues

**Figure 5:**
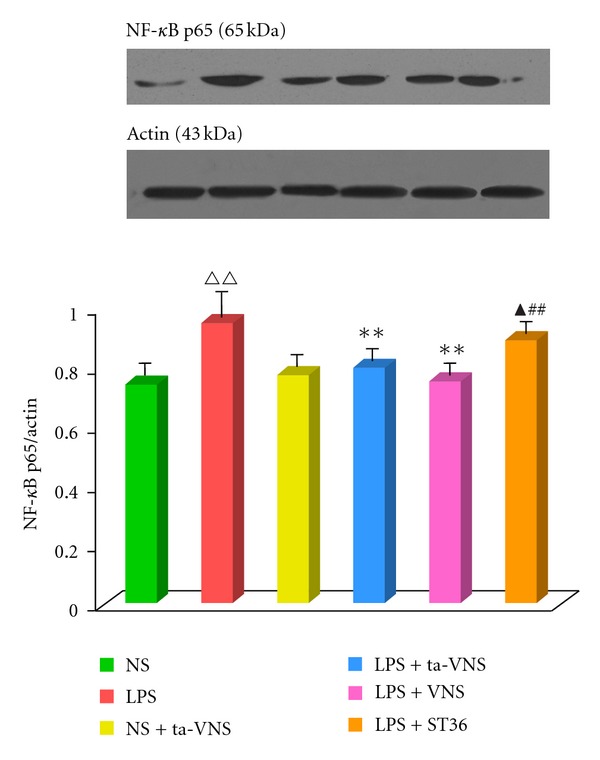
VNS or ta-VNS suppresses LPS-induced NF-*κ*B expression; ST36 stimulation did not affect NF-*κ*B in endotoxemia animals significantly. ta-VNS did not significantly affect pulmonary NF-*κ*B expression with normal saline administration. NF-*κ*B expressions were measured by western blot technique. Data are shown by mean ± SEM (*n* = 12 per group). ^∆∆^ *P* < 0.01 versus normal saline (NS) group; **P < 0.01 versus LPS group (LPS); ^##^ *P* < 0.01 versus LPS+VNS group; ^▲^ *P* < 0.05 versus LPS+ ta-VNS.

**Figure 6:**
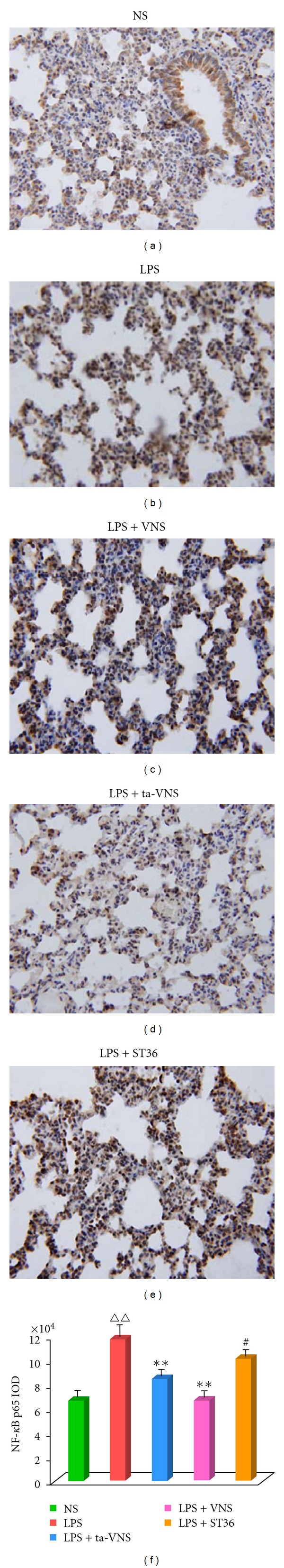
Immunohistochemical staining with anti-NF-*κ*B antibodies reveals significant decrease in LPS-induced NF-*κ*B immunoreactivity evoked by interventions as of VNS, ta-VNS, and TEAS on ST36. Data are expressed as mean ± SEM (*n* = 12 per group). ^∆∆^ *P* < 0.01 versus the normal saline (NS) group; **P < 0.01 versus LPS group (LPS); ^#^ *P* < 0.05 versus LPS+VNS group. Original magnification: ×400.

The systemic administration of LPS was followed with a significantly increased expression of NF-*κ*B p65 in lung tissues (Figures 4 and 5). Both electrical VNS and ta-VNS strongly inhibited LPS-induced NF-*κ*B p65 (*n* = 10, *P* < 0.01, Figures 4 and 5). TEAS on ST36 did not have the same effect (Figures 5 and 6).

### 3.5. Effect of ta-VNS or TEAS on ST36 on NF-*κ*B p65 Was Blunted by Vagotomy

**Figure 7:**
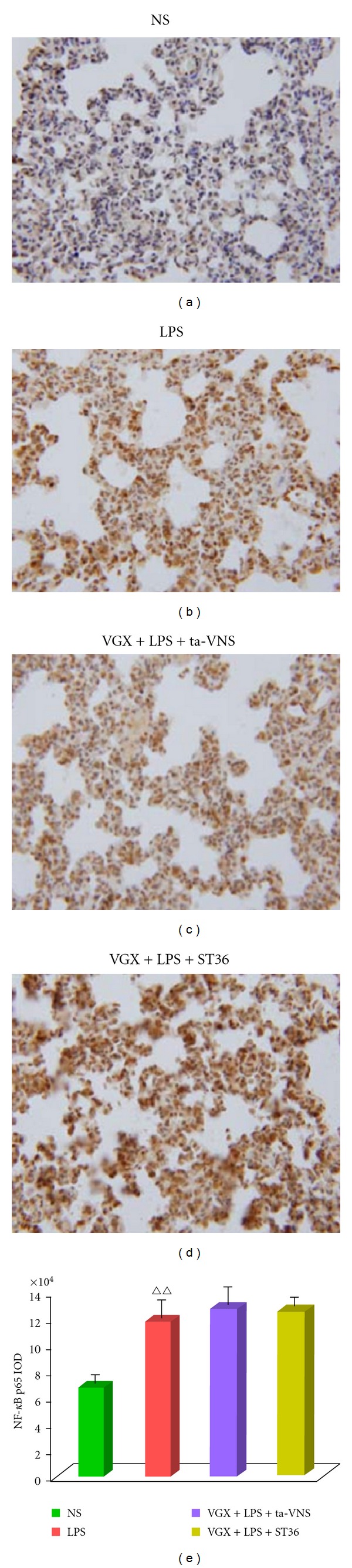
ta-VNS or ST36 stimulation with bilateral cervical vagotomy (VGX) fails to inhibit the LPS-induced overexpression of NF-*κ*B. NF-*κ*B distribution was measured by immunohistochemical staining. Data are shown as mean ± SEM (*n* = 12 per group). ^∆∆^ *P* < 0.01 versus the normal saline (NS) group. Original magnification: ×400.

We pretreated animals with vagotomy. The result indicated that ta-VNS or ST36 failed to inhibit the expressions of NF-*κ*B p65 after vagotomy (Figure 7).

## 4. Discussion

Here, we reported our original study that auricular concha stimulation is also a potent anti-inflammatory stimulus that can modulate immune factors in endotoxemia rat model. The present study demonstrates that ta-VNS may have an important role in suppressing inflammatory responses, and this contributes to the involvement of the cholinergic anti-inflammatory pathway in the mechanism. 

Previous study demonstrated that the cholinergic anti-inflammatory pathway is a *α*7nAChR-dependent, vagus nerve-mediated pathway [1]. It can inhibit macrophage activation through parasympathetic outflow, which functions as an anti-inflammatory pathway in systemic and local inflammation. Inflammatory signals stimulate sensory fibers that ascend in the vagus nerve to synapse in the NTS and then activate efferent fibers in the vagus nerve to suppress peripheral cytokine release through alpha7nAChR.

The most important cytokine involved is TNF-*α*, which activates other proinflammatory cytokines such as IL-1*β*, IL-6, and high mobility group B1 (HMGB1) and amplifies other inflammatory mediators. VNS has been demonstrated to inhibit proinflammatory cytokine production [23–25], especially the release and synthesis of TNF-*α*. The present study indicates that VNS decreases LPS-induced TNF-*α*, IL-1, and IL-6 in circulation. And ta-VNS reduced the levels of proinflammatory cytokines TNF-*α*, IL-1*β*, and IL-6, which is similar to the effect of VNS. After administration of *α*7nAChR antagonist *α*-BGT, ta-VNS failed to attenuate serum TNF-*α* level, which is consistent with previous reports [6–10, 20, 23]. This result indicated that *α*7nAChR played a critical role in anti-inflammatory effect of ta-VNS. The present study also demonstrated that vagotomy exacerbated serum TNF responses to inflammatory stimulation, sensitized animals to the lethal effects of endotoxin, and abolished the anti-inflammatory effect of ta-VNS. The results indicated that ta-VNS fails to suppress excessive cytokine response characterized by exaggerated TNF-*α* level if there is deficiency in either the *α*7nAChR subunit or vagus nerve. 

NF-*κ*B is a master transcription factor controlling the expression of a wide range of proinflammatory genes [26–28]. Previous studies reported that NF-*κ*B is involved in TNF-*α* genetic activation and TNF-*α* production [29, 30]. In the present study, both western blot data and immunehistochemical results indicated that ta-VNS suppresses the LPS-induced NF-*κ*B expression in rat lung tissue (Figures 4 and 5), which mimicking the effects of VNS [25, 31]. In vagotomy animals, ta-VNS failed to inhibit increased NF-*κ*B expression, suggesting ta-VNS functions in situations that require intact vagus nerve. 

Some investigators demonstrated that electroacupuncture (EA, on ST36, PC6, and GV20) could increase the vagal activity of experimental animals and human subjects [13–17, 32–34]. The present study demonstrated that TEAS on ST36 decreased serum TNF-*α* level in endotoxemia rats. After pretreatment with vagotomy or *α*7nAChR antagonist *α*-BGT, TEAS failed to inhibit serum proinflammatory level in LPS-induced endotoxemia animals. 

Auricular acupuncture, as a special form of acupuncture, has been used for the treatment of different disorders for centuries in China. Our research group previously demonstrated that auricular acupuncture stimulation could activate neurons of NTS and upregulate vagal tune, to decrease MAP and HR [35], to trigger gastric motility [36]. Our previous studies also demonstrated that TEAS of auricular concha could activate the parasympathetic nervous system and mimic the effect of VNS to suppress epileptic seizures [18]. In the present study, the results showed that ta-VNS inhibited proinflammatory cytokine levels and suppressed NF-*κ*B expressions in endotoxaemia rats (Figures 1, 4, and 5), which is similar to the effect of VNS. However, vagotomy or *α*7nAChR antagonist *α*-BGT could diminish the effect of ta-VNS on the anti-inflammatory responses, suggesting that auricular acupuncture may perform an anti-inflammatory effect via cholinergic anti-inflammatory pathway. 

**Figure 8:**
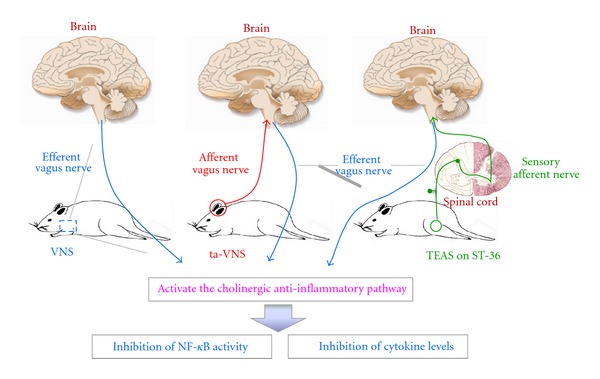
The anti-inflammatory mechanisms of the three interventions used in the present study might be as follows: (1) VNS directly activates the cholinergic anti-inflammatory pathway via stimulating efferent vagus nerve. (2) ta-VNS evoked the activity of the auricular branch of vagus nerve (ABVN). The activated signals ascending within afferent vagus nerve are transmitted to the nucleus tractus solitarius. The integrated output is carried by the efferent vagus nerve to inhibit inflammatory responses. (3) TEAS on ST36 activates the somatic fiber endings of the skin around ST36 point, sending signals to the spinal cord via somatic sensory nerve fibers. The nerve impulses were relayed and integrated by NTS by the secondary order neurons in the spinal cord, and the cholinergic anti-inflammatory pathways are activated by the increased efferent vagal output.

In general (Figure 8), VNS directly activates the cholinergic anti-inflammatory pathway via stimulating efferent vagus nerve. As the peripheral branch [18], auricular branch of vagus nerve (ABVN) innervates the auricular concha and the external auditory meatus. Stimulation of the ABVN region could evoke parasympathetic excitation [37–39]. Acupuncture in the area of auricular concha may increase discharge of NTS [18], as the central terminal nuclear for afferent vagal fibers, which primarily transmit signals from local inflammation lesion [4, 40]. Thus, we hypothesize that ABVN could be evoked by ta-VNS, and the activated signals ascend with vagal input to the NTS. The signals are processed within the NTS, and the integrated output signal is carried by efferent vagus nerve to inhibit inflammatory responses. TEAS on the acupoint of ST36 activates the somatic fiber endings around ST36 point, which send the acupuncture signals to the spinal cord via somatic sensory nerve fibers. In the spinal cord, the nerve impulses are delivered to the NTS by the secondary order neurons, where the signals were processed. Ultimately, the increased efferent vagal output activates the cholinergic anti-inflammatory pathway.

## 5. Conclusions

The results presented here demonstrate that ta-VNS plays an important role in immuneregulation, through the activation of the cholinergic anti-inflammatory pathway and the downregulation of proinflammatory cytokine expressions and NF-*κ*B activities. VNS and TEAS on ST36 might suppress the inflammatory responses via different mechanisms.

## Abbreviations

ta-VNS: Transcutaneous auricular vagus nerve stimulation
LPS: lipopolysaccharide
NTS: Nucleus tractus solitarius
DMN: Dorsal motor nucleus of the vagus
VNS: Vagus nerve stimulation
TEAS: Transcutaneous electric acupoint stimulation
Ach: Acetylcholine
*α*7nAChR: Nicotinic acetylcholine receptors
NF-*κ*B p65: NF-kappa B p65
TNF-*α*: Tumour necrosis factor-alpha
IL-1*β*: Interleukin-1 beta
IL-6: Interleukin-6
*α*-BGT: *α*-Bungarotoxin
NS: Normal saline
VGX: Vagotomy
ABVN: Auricular branch of vagus nerve.
